# Branches of Deception: A Tree-in-Bud Pattern Revealing Advanced Non-small Cell Lung Cancer (NSCLC)

**DOI:** 10.7759/cureus.90957

**Published:** 2025-08-25

**Authors:** Chetana R Pendkar

**Affiliations:** 1 Pulmonary and Critical Care, Bellin Memorial Hospital, Green Bay, USA

**Keywords:** bronchoalveolar lavage, lung cancer, mucinous adenocarcinoma, pulmonary infection mimic, tree-in-bud opacities

## Abstract

The tree-in-bud (TIB) opacities pattern seen on chest CT scans, characterized by small, branching, nodular opacities, primarily indicates diseases of the small airways, particularly bronchiolitis. We present a rare differential diagnosis for this CT finding.

A 46-year-old female patient with asthma and environmental smoke exposure presented with subacute cough and dyspnea. Chest CT revealed diffuse TIB opacities of the left lung and mediastinal lymphadenopathy. Initial suspicion was for atypical infection. However, bronchoscopy with bronchoalveolar lavage (BAL), brushing, and lymph node FNA revealed atypical and malignant cells. Immunohistochemistry showed tumor cells positive for TTF-1 and negative for p40, confirming the diagnosis of pulmonary adenocarcinoma. Ki-67 highlighted increased proliferative activity. The final diagnosis was stage IIIc non-small cell lung cancer (NSCLC). The patient was treated sequentially with platinum-doublet chemotherapy and multiple systemic therapies, including crizotinib (discontinued due to toxicity) and durvalumab. She is currently on maintenance pembrolizumab for palliative care, with stable disease and improved functional status.

This case illustrates how mucinous adenocarcinoma can present with imaging features mimicking infection. Misdiagnosis can delay appropriate treatment. Early cytologic evaluation and immunohistochemistry were key to diagnosis. In high-risk patients with persistent TIB opacities unresponsive to antimicrobials, malignancy should be considered. Prompt tissue sampling and immunophenotyping can prevent diagnostic delay and inform timely management.

## Introduction

Lung non-mucinous adenocarcinoma, the most common subtype of non-small cell lung cancer (NSCLC), typically presents as a solitary pulmonary nodule or mass on imaging. Diagnosis is often straightforward when classical radiologic features and clinical symptoms align. However, atypical presentations can lead to diagnostic delays, particularly when imaging mimics benign or infectious etiologies [1,2]. One such radiographic finding is the tree-in-bud (TIB) pattern, characterized by centrilobular nodules with branching linear opacities. This pattern is most commonly associated with infectious bronchiolitis, atypical mycobacterial infection, or airway-centered infectious and inflammatory conditions [1].

The presence of TIB opacities is a high risk for significant underlying lung pathology, such as atypical mycobacterial infections, bacterial bronchopneumonia, and aspiration. While rare, primary pulmonary malignancies may present with TIB-like features due to tumor spread along airways, mucin impaction, or lymphangitic carcinomatosis [3].

This case report describes a diagnostic challenge involving a middle-aged woman with asthma and recent environmental smoke exposure whose presumed infectious TIB pattern was ultimately revealed to be advanced-stage pulmonary adenocarcinoma.

## Case presentation

A 46-year-old caucasian woman, a non-smoker, with a history of moderate persistent asthma and recent environmental smoke exposure, presented with three weeks of progressive dyspnea and productive cough. She denied fever, hemoptysis, or weight loss. Physical examination was unremarkable, and oxygen saturation was normal on room air.

**Figure 1:**
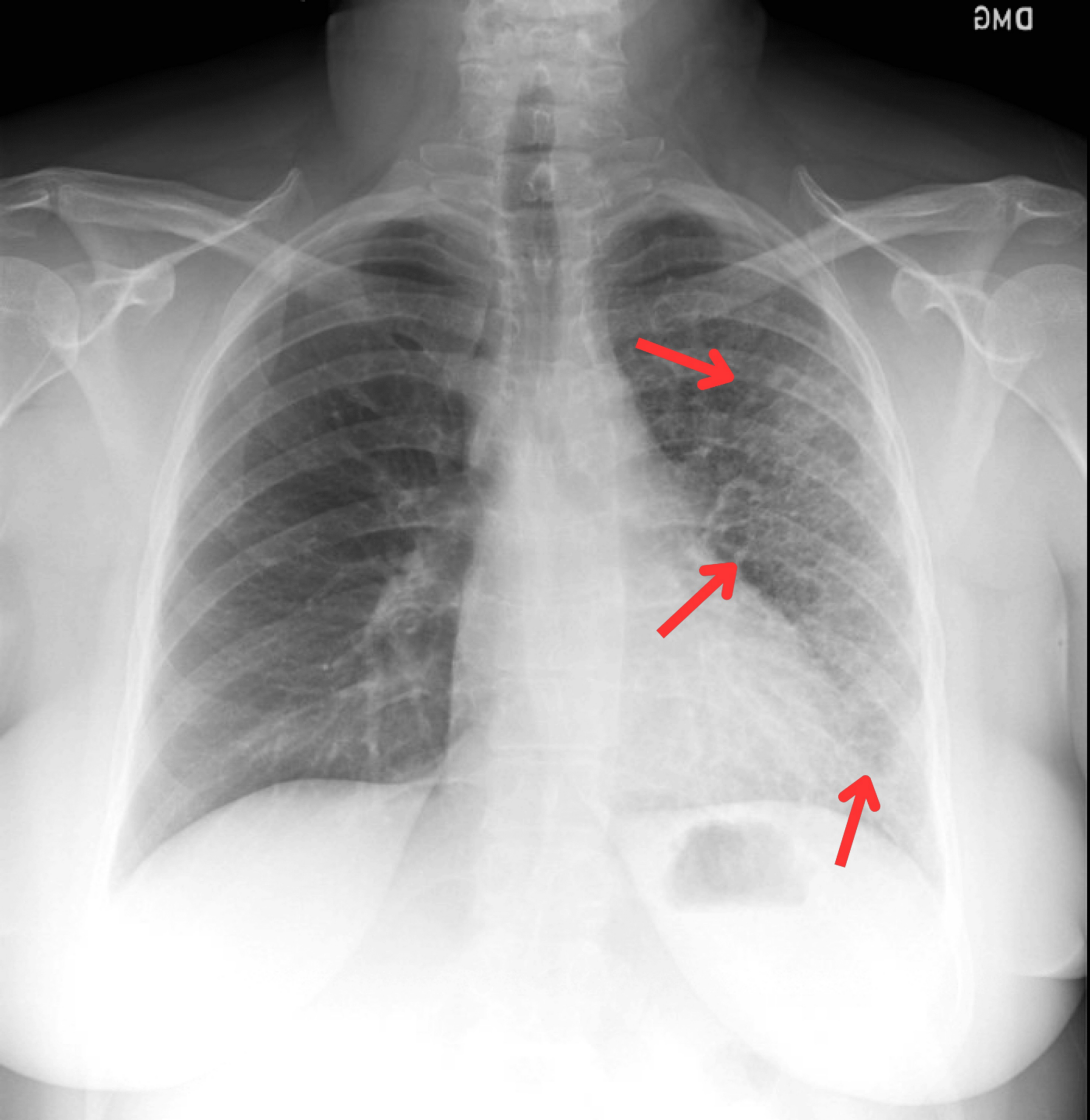
Chest radiograph (posteroanterior view) showing subtle hazy opacities and reticulonodular markings with volume loss predominantly in the upper and mid zones of the left lung. These findings were initially interpreted as consistent with atypical infection or inflammatory bronchiolitis. The red arrows represent increased reticular markings.

An initial chest radiograph (Figure 1) demonstrated subtle hazy opacities with reticulonodular markings more pronounced in the left upper and mid zones. These findings were initially interpreted as suggestive of atypical infection or inflammatory bronchiolitis.

**Figure 2:**
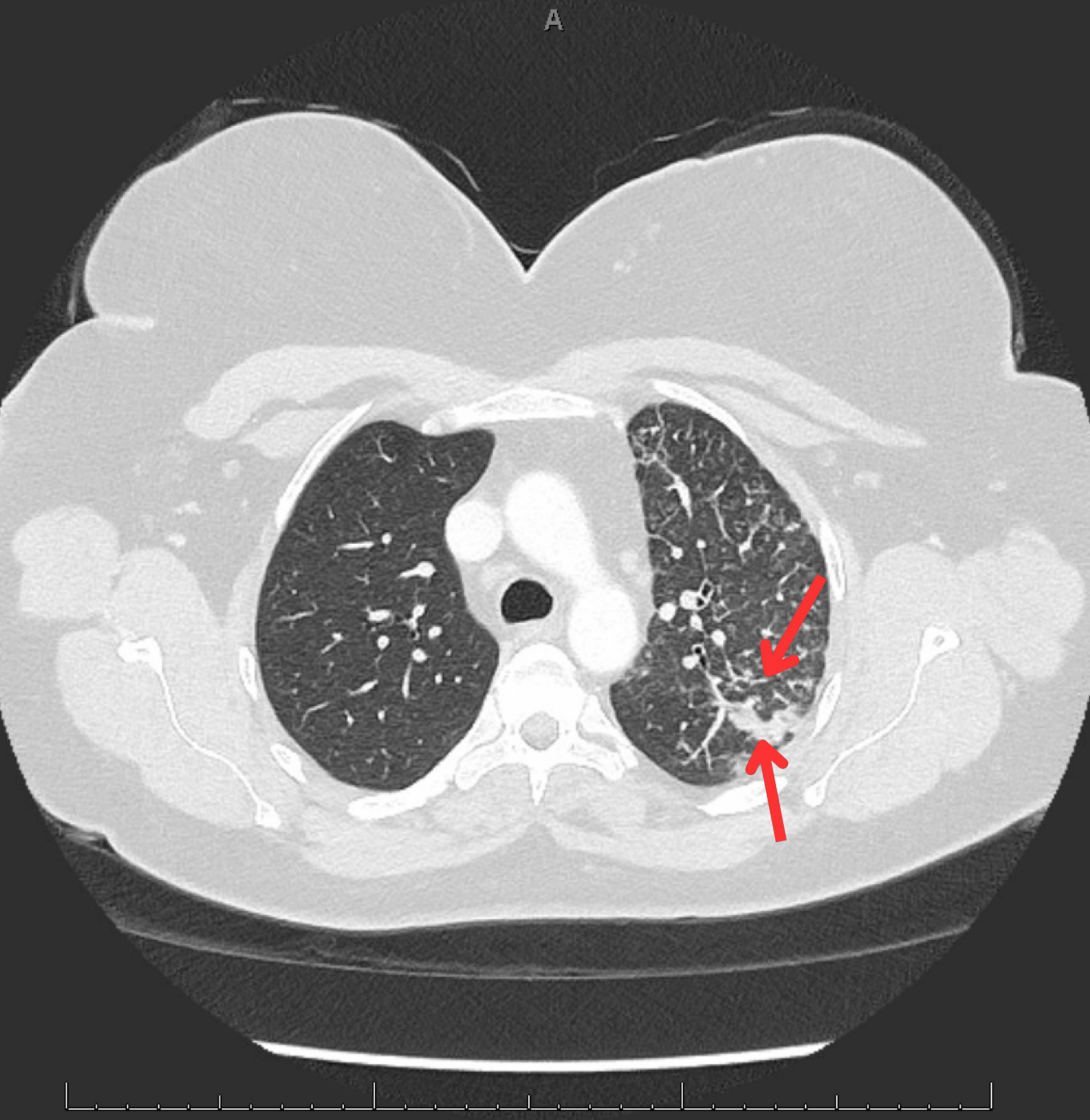
Axial CT image showing tree-in-bud opacities along with consolidative and nodular changes in the upper lobe of the left lung. The red top arrow shows linear opacity with distal airway plugging, and the bottom red arrow shows consolidative changes in the upper lobe of the left lung.

**Figure 3:**
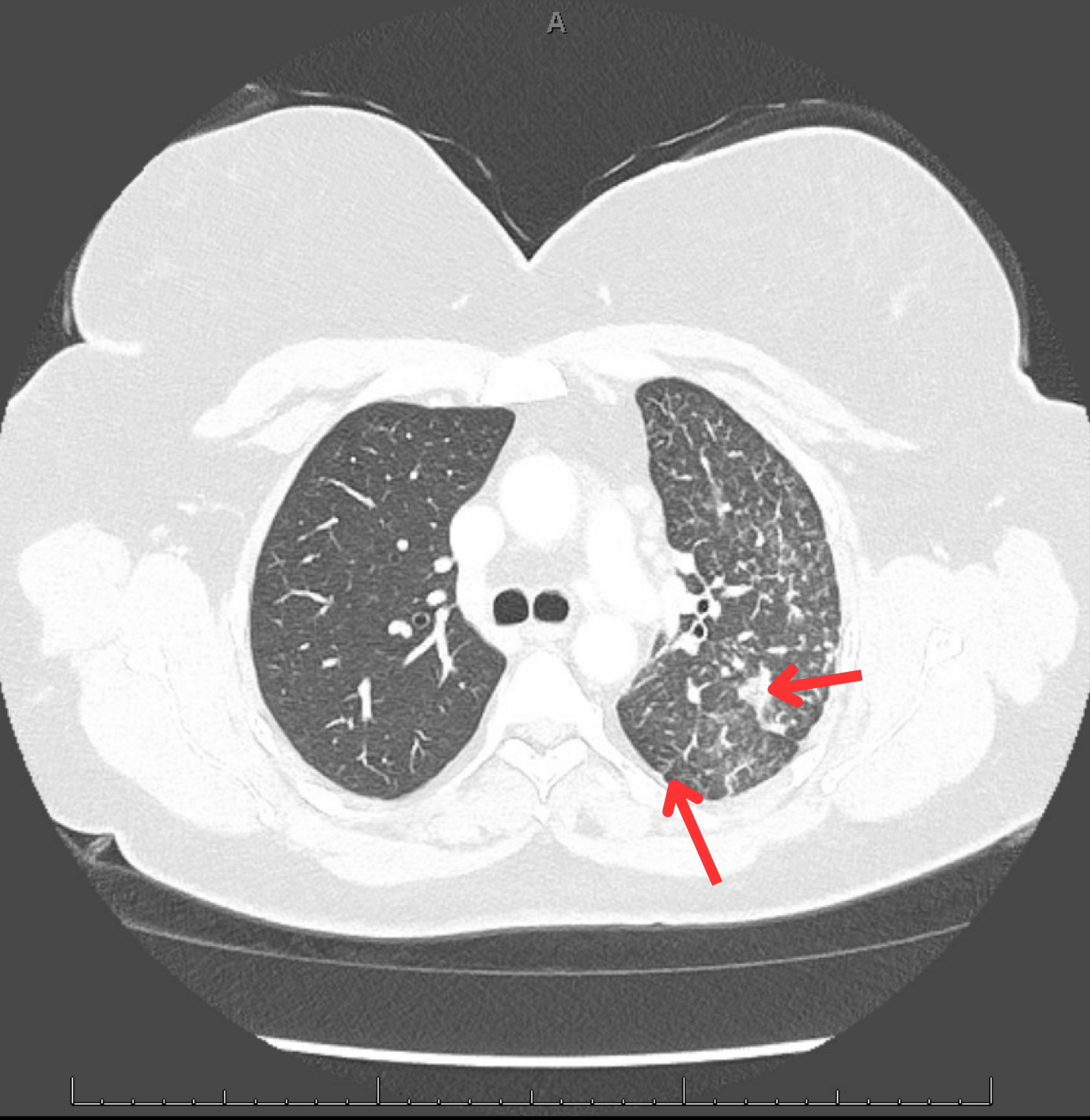
Axial CT image showing nodular infiltrates and interstitial thickening in the left lung showing patchy nodular infiltrates and interstitial thickening within the left lung. The opacities are predominantly peribronchiolar and subpleural in distribution, contributing to diagnostic ambiguity between infection and malignancy. The top red arrow indicates consolidative change, and the bottom red arrow shows linear opacity with distal airway plugging.

**Figure 4:**
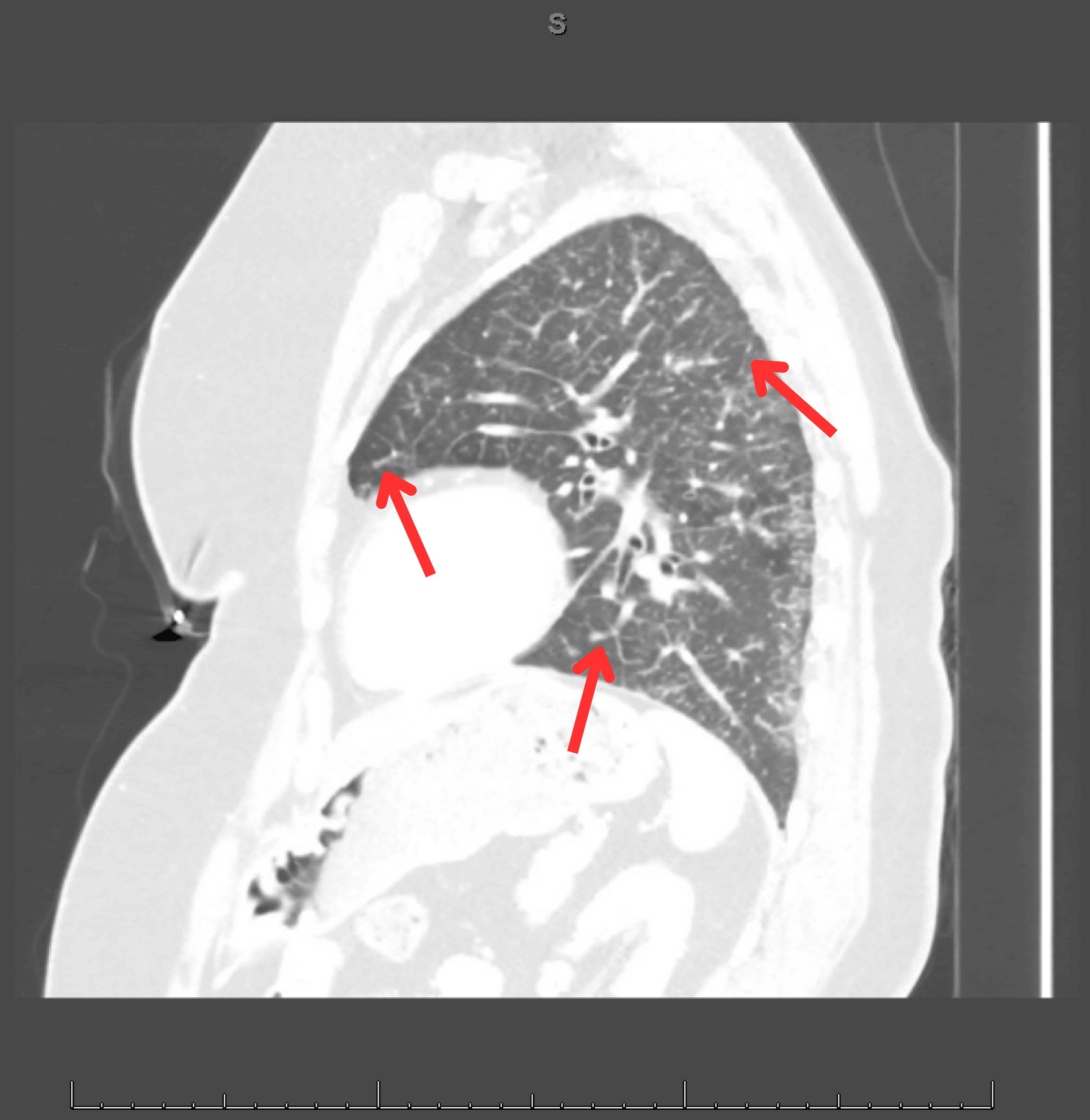
Sagittal reconstruction of the chest CT. The linear opacities and nodular thickening further support the tree-in-bud appearance. All red arrows indicate linear opacities due to small airway thickening and bronchiolar plugging.

Follow-up contrast-enhanced chest computed tomography (CT) revealed extensive centrilobular nodules, tree-in-bud opacities, and reticulonodular thickening diffusely affecting the left upper and lower lobes (Figures 2, 3) [1]. A sagittal CT view (Figure 4) further demonstrated subpleural and peribronchiolar opacities with preserved lung volume. Enlarged mediastinal and hilar lymph nodes were present bilaterally. The initial impression was bronchiolar infectious or inflammatory disease.

Due to a lack of response to antibiotics, the patient was investigated with a bronchoscopy. Bronchoscopy with bronchoalveolar lavage (BAL) and brushing was performed in the left lower lobe (LLL). Endobronchial ultrasound-guided fine needle aspiration (FNA) was carried out on multiple mediastinal and hilar lymph node stations, including 4L, 4R, 7, 11L, and 11R. Cytology from the left lower lobe revealed atypical, cohesive epithelial cells with enlarged nuclei and visible nucleoli. Cytology confirmed adenocarcinoma in stations 4L, 4R, and 7, while 11L and 11R demonstrated atypical cells. Immunohistochemistry showed strong positivity for TTF-1 and AE1/AE3, and negativity for p40, confirming pulmonary adenocarcinoma. Ki-67 demonstrated increased proliferative activity.

**Figure 5:**
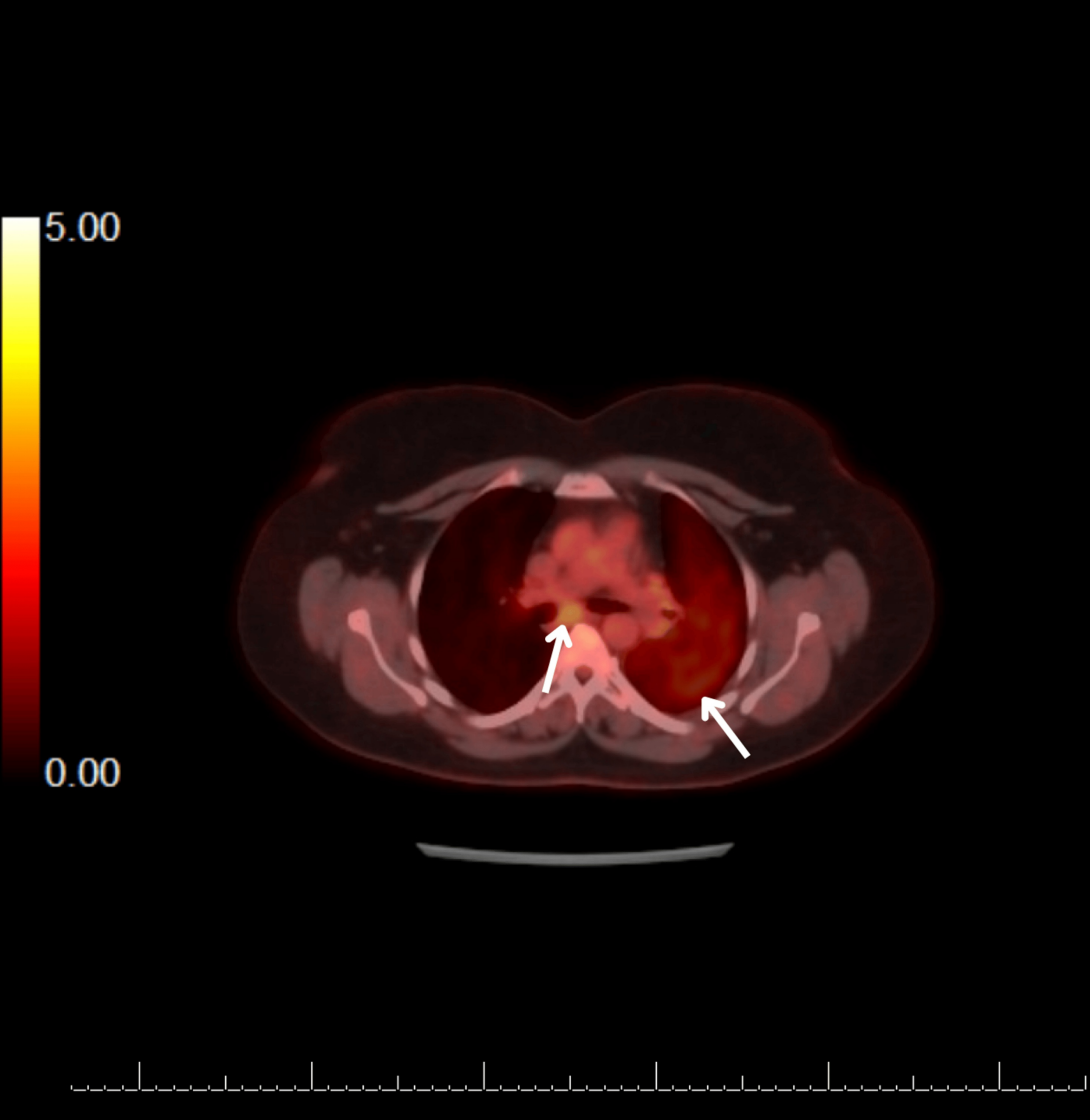
PET-CT image showing FDG-avid uptake in the left lung and mediastinal lymph nodes. FDG: fludeoxyglucose

Staging PET-CT demonstrated moderate fludeoxyglucose (FDG) uptake in the left lung consolidation and mediastinal nodes, consistent with metabolically active malignancy (Figure 5). The final diagnosis was stage IIIc (T4, N3, M0) non-small cell lung cancer (NSCLC) with multi-station nodal involvement [4]. Molecular profiling revealed no actionable epidermal growth factor receptor (EGFR) mutations or anaplastic lymphoma kinase (ALK) rearrangements, and PD-L1 expression was reported at 30%.

The patient began first-line platinum-doublet chemotherapy with cisplatin, pemetrexed, and pembrolizumab. She later transitioned to carboplatin-based regimens, then crizotinib (discontinued due to toxicity), followed by durvalumab. She is currently on maintenance pembrolizumab and remains clinically stable.

## Discussion

NSCLC is the most common form of lung cancer, with adenocarcinoma being the predominant histologic subtype. Typically, it presents as a solitary pulmonary nodule or mass. However, rare presentations can mimic infectious or inflammatory diseases, delaying accurate diagnosis and timely treatment [1,3]. This case illustrates a diagnostically challenging scenario in which pulmonary adenocarcinoma presented with TIB opacities, a radiologic pattern more commonly associated with infectious bronchiolitis or airway-centered inflammatory processes [1,2].

The TIB pattern refers to centrilobular nodules connected to linear branching structures resembling budding trees. It is typically interpreted as a sign of endobronchial spread of infection, particularly mycobacterial or fungal etiologies, or aspiration [1]. In this patient, the radiologic findings were confined to the left lung, correlating with her symptoms and prior asthma diagnosis, further reinforcing the assumption of infection. However, her lack of clinical improvement despite antibiotics prompted further evaluation.

CT findings included reticulonodular opacities, interlobular septal thickening, and mediastinal lymphadenopathy, raising concern for alternative diagnoses. Bronchoscopy with cytology and immunohistochemistry proved diagnostic, demonstrating cohesive, atypical epithelial cells positive for TTF-1 and negative for p40-findings consistent with pulmonary adenocarcinoma. The Ki-67 positivity indicated increased tumor proliferation. These results highlight the importance of early cytological evaluation in cases of non-resolving pulmonary infiltrates, even when radiologic findings suggest an infectious etiology [3].

This case adds to a small but growing body of literature describing NSCLC masquerading as TIB opacities. Raju et al. [1] previously highlighted that such imaging features, though uncommon in malignancy, may occur due to mucin-producing tumor cells obstructing bronchioles, lymphatic tumor spread, or tumor emboli. In our patient, the radiologic mimicry likely resulted from a combination of tumor spread along alveolar and peribronchiolar pathways and accompanying lymphangitic carcinomatosis.

Beyond the diagnostic challenge, this case also reflects the evolving landscape of NSCLC treatment. Following diagnosis, our patient was treated with multiple lines of therapy, including platinum-doublet chemotherapy and immunotherapy. Although she was not eligible for targeted therapy due to the absence of actionable mutations such as ALK or EGFR, her tumor’s PD-L1 expression of 30% supported the use of immune checkpoint inhibitors. While durvalumab was initially employed, she eventually transitioned to maintenance pembrolizumab as part of a palliative care strategy. Her clinical stability and response highlight the potential benefits of immunotherapy in patients with PD-L1-positive disease [5].

While TIB opacities are predominantly linked to infectious or inflammatory causes, the incidence of underlying malignancy presenting with this pattern is not well quantified in current literature. Most available evidence stems from isolated case reports or small case series, with no large-scale studies offering prevalence estimates. This knowledge gap presents a diagnostic challenge, particularly in patients who are not responding to antimicrobial therapy. Our case adds to this limited pool of data and underscores the need for heightened clinical vigilance. In patients with persistent TIB findings - especially those with risk factors such as smoking history, environmental exposures, or systemic symptoms - a thorough diagnostic workup, including cytology and tissue sampling, should be considered to rule out malignancy.

## Conclusions

This case highlights the diagnostic complexity of pulmonary adenocarcinoma presenting with tree-in-bud opacities - a radiologic pattern more commonly associated with infection. Clinicians should maintain a broad differential when clinical and imaging findings diverge, particularly in high-risk patients unresponsive to empiric therapy. Early tissue sampling and immunohistochemical evaluation are critical to avoid diagnostic delays. While treatment details are secondary, the patient’s clinical trajectory reinforces the importance of timely diagnosis in guiding appropriate care.
